# The complete mitochondrial genome of *Hemigrapsus penicillatus* (De Haan, 1835) (Decapoda, Varunidae)

**DOI:** 10.1080/23802359.2018.1443037

**Published:** 2018-02-23

**Authors:** Mustafa Zafer Karagozlu, Jung-Il Kim, Tae-June Choi, Thinh Do Dinh, Chang-Bae Kim

**Affiliations:** a Department of Biotechnology, Sangmyung University, Seoul, Korea;; b Institute of Marine Environment and Resources, Vietnam Academy of Science and Technology, Haiphong, Vietnam

**Keywords:** Decapoda, Grapsoidea, Varunidae, complete mitogenome, *Hemigrapsus penicillatus*

## Abstract

*Hemigrapsus penicillatus* is a small grapsoid crab which is found in Japan, Taiwan, Korea, and China coasts. In this study a complete mitogenome of Korean *H. penicillatus* was analyzed and phylogenetic relationships in the family Varunidae were investigated. The mitogenome size is 16,486 bp with 34.1% A, 18.1% C, 11.4% G, and 36.4% T nucleotide distributions. Genome structure and gene orientations are identical with previous records from the family and mitochondrial protein-coding gene based phylogenetic tree suggested that the closest species to *H. penicillatus* is *H. sanguineus*. This is the second complete mitogenome record from the genus *Hemigrapsus* and the first record for the species.

Varunidae is a thoracotrematan crab family which consists of 40 genera (WoRMS Editorial Board 2018). Previously, the members of this family were placed at the rank of subfamily in the Grapsidae but their location in the phylogenetic tree is revised and they are reorganized under the family Varunidae (Schubart et al. 2006). Despite genus richness in the family, there are only 13 complete mitogenomes recorded from five different genera. In the present study, complete mitochondrial genome of a Varunidae species *Hemigrapsus penicillatus* was analyzed and phylogenetic relationship of the species were investigated due to the complete mitgenome information.

The *H. penicillatus* specimen was collected from south coastal sea of South Korea, Namhae-gun, Gyeongsangnam-do (34° 52′ 23″ N, 127° 52′ 7″ E) and identified by COI barcoding. NGS sequencing was subjected to the gDNA (Miseq, Illumina, San Diego, CA, USA), and paired end reads of mitogenome sequences were assembled and annotated using MITObim (Hahn et al. 2013) and MITOS (Bernt et al. 2013), respectively. Phylogenetic tree was reconstructed based on the concatenated amino acid sequences of 13 mitochondrial protein-coding genes using the software MEGA 7.0 (Kumar et al. 2016). The specimen was stored in Department of Biotechnology, Sangmyung University, Korea (SM00239).

The size of the mitogenome is 16,486 bp (GenBank accession number: MG751772), which is the longest mitogenome record from the family Varunidae. The mitogenome has 13 protein-coding genes, 22 tRNAs, two rRNAs, and one putative control region with 34.1% A, 18.1% C, 11.4% G, and 36.4% T nucleotide distributions. Previously, complete mitogenome of *H. sanguineus* (KX456205) was recorded from the same genus. In comparison to the mitogenomes, the *H. penicillatus* mitogenome is approximately 200 bp longer. Despite size difference, structure and gene orientations in both the mitogenomes are identical. The main difference between two mitogenome is the size of the control region, which is located between *tRNA-Val* and *tRNA-Gln*. The control region of the *H. penicillatus* mitogenome is approximately 200 bp longer, which explains the size difference between the mitogenomes. Therewithal A–T content of control regions is slightly different. In *H. penicillatus*, mitogenome control region has 76.2% A–T content while *H. sanguineus* mitogenome control region has 80.7% A–T.

Phylogenetic tree of the family Varunidae was reconstructed to investigate phylogenetic relationship of *H. penicillatus* in the family (Figure 1). The reconstruction of the phylogenetic tree suggested that *H. sanguineus* was the closest species to the *H. penicillatus* and the clade which included *Hemigrapsus* species was early branched than the other genera of the family. Similar results declared by mitochondrial ribosomal RNA gene (16S rRNA)-based study (Schubart et al. 2001) and combination of mitochondrial small and large ribosomal subunit genes (12S and 16S rRNA)-based study (Schubart et al. 2006). Although the phylogeny of Grapsoidea was revised by molecular phylogeny studies, the phylogenetic relationship of Grapsoidea had not been fully understood due to limited genomic data records. This study provides additional data for the family Varunidae phylogeny. For further studies complete mitochondrial genome data records should be increased to understand the phylogenetic relationships.

**Figure 1.**
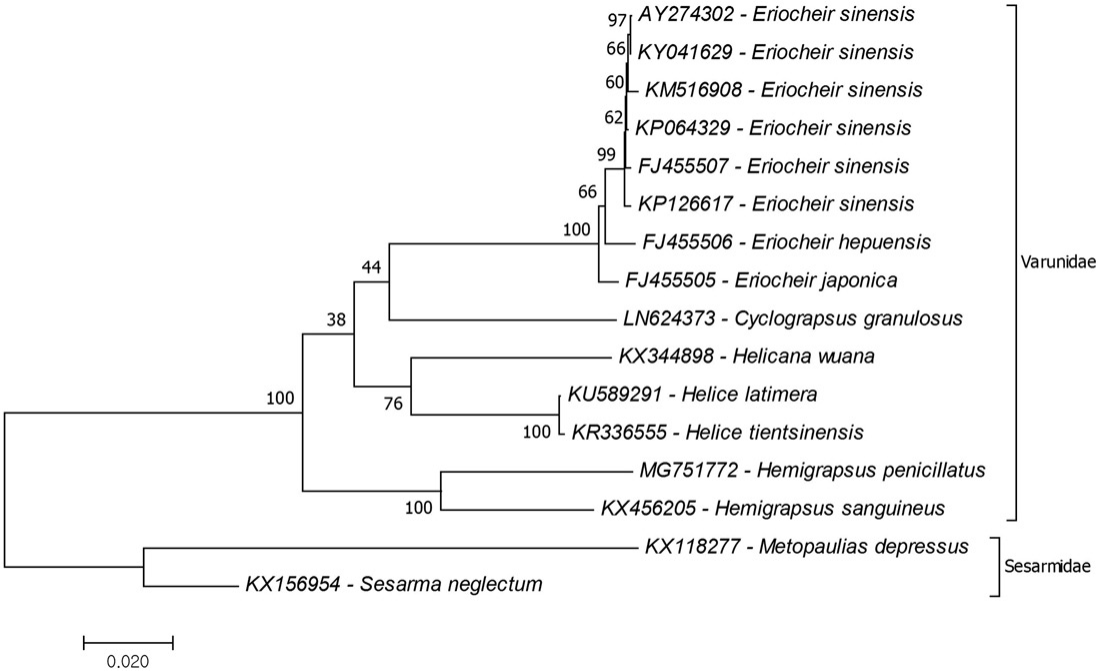
Phylogenetic relationship of Varunidae species evaluated due to mitochondrial protein coding genes. The complete mitochondrial genome of the *H. penicillatus* (MG751772) was provided by the present study and the remaining mitogenome data were retrieved from the GenBank. The two species from the family Sesarmidae represent outgroup.
